# Testosterone Replacement Therapy and Risk of COVID-19 and Effect of COVID-19 on Testosterone's Treatment Effect

**DOI:** 10.1210/jendso/bvaf002

**Published:** 2025-02-05

**Authors:** Karol M Pencina, A Michael Lincoff, Eric A Klein, Steven E Nissen, Yili Valentine Shang, Nader Khan, Xue Li, Anna Chan, Michael G Miller, Shalender Bhasin

**Affiliations:** Research Program in Men's Health: Aging and Metabolism, Boston Claude D. Pepper Older Americans Independence Center, Brigham and Women's Hospital, Harvard Medical School, Boston, MA 02115, USA; Cleveland Clinic Coordinating Center for Clinical Research (C5Research), Department of Cardiovascular Medicine, Cleveland Clinic, Cleveland, OH 44106, USA; Cleveland Clinic Coordinating Center for Clinical Research (C5Research), Department of Cardiovascular Medicine, Cleveland Clinic, Cleveland, OH 44106, USA; Department of Urology, Glickman Urological and Kidney Institute, Cleveland Clinic, Cleveland, OH 44106, USA; Cleveland Clinic Coordinating Center for Clinical Research (C5Research), Department of Cardiovascular Medicine, Cleveland Clinic, Cleveland, OH 44106, USA; Research Program in Men's Health: Aging and Metabolism, Boston Claude D. Pepper Older Americans Independence Center, Brigham and Women's Hospital, Harvard Medical School, Boston, MA 02115, USA; AbbVie Inc., North Chicago, IL 60064, USA; AbbVie Inc., North Chicago, IL 60064, USA; AbbVie Inc., North Chicago, IL 60064, USA; AbbVie Inc., North Chicago, IL 60064, USA; Research Program in Men's Health: Aging and Metabolism, Boston Claude D. Pepper Older Americans Independence Center, Brigham and Women's Hospital, Harvard Medical School, Boston, MA 02115, USA

**Keywords:** testosterone, testosterone treatment and risk of COVID-19, COVID-19 and response to testosterone, hypogonadism and risk of COVID-19

## Abstract

**Context:**

Whether circulating testosterone, dihydrotestosterone, and estradiol levels or testosterone replacement therapy (TRT) affects the risk of COVID-19 and whether COVID-19 affects response to TRT remains unknown.

**Objective:**

The study evaluated whether baseline testosterone, dihydrotestosterone, and estradiol levels or TRT are associated with risk of developing COVID-19 and whether COVID-19 affects treatment response to TRT.

**Methods:**

Among 5204 men, aged 45 to 80 years, with hypogonadism in the TRAVERSE trial, 379 developed COVID-19. We compared baseline and on-treatment hormone levels, and safety and efficacy in participants with and without COVID-19 diagnosis.

**Results:**

Neither baseline nor on-treatment testosterone, estradiol, and dihydrotestosterone levels prior to COVID-19 differed significantly between men with and without COVID-19 diagnosis. Incidence of COVID-19 was similar in participants randomized to TRT or placebo groups (3-year Kaplan-Meier incidence 8.0% in TRT and 8.6% in placebo group, *P* = .823). Incidences of COVID-19-related hospitalizations (38.5% vs 32.8%, *P* = .222) and deaths (12.8% vs 8.9%, *P* = .247) were similar in the TRT and placebo groups. Changes in hypogonadal symptoms, libido, energy, and hemoglobin/hematocrit in response to TRT were attenuated in testosterone-treated men who developed COVID-19. Incidences of major adverse cardiovascular events, venous thromboembolism, and acute kidney injury were similar in those with COVID-19 diagnosis and those without.

**Conclusion:**

In men with hypogonadism and cardiovascular disease (CVD) or increased risk of CVD, baseline and pre-COVID-19 on-treatment testosterone, dihydrotestosterone, and estradiol levels were similar in those who developed COVID-19 and those who did not. TRT did not affect the risk of COVID-19. COVID-19 attenuated the treatment response to TRT.

Men are at increased risk of coronavirus disease 2019 (COVID-19) [1-3]; however, the mechanisms underlying these sex differences in the risk of COVID-19 remain incompletely understood. Surgical orchiectomy in C57BL6 mice is associated with reduced expression levels of angiotensin converting enzyme 2 (*Ace2*) in lung epithelial cells [4], the receptor for the severe acute respiratory syndrome coronavirus 2 (SARS-CoV-2); conversely, testosterone increases ACE2 expression in airway smooth muscle cells from men [5]. Testosterone also regulates the expression of transmembrane protease serine 2 (*Tmprss2*) [6, 7], which is involved in the cleavage and activation of SARS-CoV-2 in lung epithelial cells. Montopoli et al [8] reported that men with prostate cancer rendered hypogonadal by androgen deprivation therapy (ADT) were protected from SARS-CoV-2 infection. Subsequent observational studies of men receiving ADT for prostate cancer yielded conflicting results; some studies reported a reduced incidence and severity of COVID-19 in men receiving ADT while others found no difference in infection risk [9-11]. Retrospective reviews of medical records and cohort studies also reported conflicting data [12-15]. One study reported preexisting hypogonadism to be associated with an increased risk of COVID-19 [13] while in an analysis of the UK Biobank data higher premorbid testosterone levels were not associated with increased risk of COVID-19 but were associated with an increased risk of COVID-19-related mortality [15]. Retrospective analyses of the effects of testosterone replacement therapy (TRT) of men with hypogonadism on the risk of SARS-CoV-2 infection also have yielded inconsistent results [13, 14]. Also, previous studies that have reported testosterone levels in relation to the risk of COVID-19 were conducted in patient care settings and used immunoassays of varying quality under non-standardized conditions. These studies suffer from the limitations inherently associated with the review of electronic medical records data. No prospective randomized trial has evaluated the effects of TRT on the risk of infection. It also remains unknown whether COVID-19 affects the efficacy response to TRT in men with hypogonadism.

The Testosterone Replacement therapy for Assessment of long-term Vascular Events and efficacy ResponSE in hypogonadal men (TRAVERSE) study, a randomized, placebo-controlled trial, was designed to determine the cardiovascular safety of TRT in middle-aged and older men with hypogonadism. The trial's design [16] and primary safety and efficacy data have been reported [14, 17-21]. The COVID-19 pandemic ensued in the middle of the trial, and 379 study participants were diagnosed with COVID-19. The occurrence of confirmed COVID-19 cases in the TRAVERSE trial offered an opportunity to address several questions unanswered by previous retrospective and observational studies. Are premorbid levels of sex hormones—testosterone, dihydrotestosterone, and estradiol—associated with the risk of developing COVID-19? Does TRT of men with hypogonadism affect the risk of developing COVID-19 disease? We also assessed whether the occurrence of COVID-19 disease had a sustained effect on efficacy endpoints such as sexual function, mood, and energy in the testosterone and placebo-treated men during the year after developing COVID-19. Moreover, as COVID-19 is associated with an increased risk of major adverse cardiovascular events (MACE), venous thromboembolism [22], cardiac arrythmias, and acute kidney injury, we assessed whether COVID-19 affects the risk of these adverse events in men receiving TRT.

## Methods

### Study Population

The analyses were performed on the full analysis set of the TRAVERSE study, which included all 5204 randomized participants. The eligibility criteria and the trial design have been published [16, 17]. Briefly, men, 45 to 80 years of age, with preexisting cardiovascular disease (CVD) or increased CVD risk, who reported one or more symptoms of hypogonadism and had 2 fasting morning serum testosterone levels less than 300 ng/dL measured using liquid chromatography tandem–mass spectrometry (LC-MS/MS), were eligible for this trial. Patients with congenital or acquired severe hypogonadism, a history of prostate cancer or undiagnosed prostate nodules, screening prostate-specific antigen (PSA) level > 3 ng/mL, thrombophilia, or uncontrolled heart failure were excluded from the study.

As described previously [16, 17], the eligible participants were randomized in 1:1 ratio to receive either 1.62% transdermal testosterone gel or matching placebo gel daily for the duration of the trial; the randomization was stratified by preexisting CVD.

### Study Assessments

The diagnosis of COVID-19 was self-reported by participants and ascertained from the adverse events listings. Because the pandemic was unanticipated, prospective testing of all participants for COVID-19 was not performed.

As described previously [16], serum testosterone levels were measured at baseline, during weeks 2, 4, 12, and 26 and during months 12, 18, 24, 36, 48, 60, and end of study. Serum dihydrotestosterone and estradiol levels were measured 24 hours after the testosterone gel application at baseline and during months 12, 24, 36, 48, 60, and end of study. Hemoglobin and hematocrit were measured at baseline, and during months 6, 12, 18, 24, 36,48, 60, and end of study. Questionnaires were administered at baseline and during months 6, 12, 24, 36, 60, and end of study [16, 19, 20]. The primary endpoint of the parent trial was MACE, defined as time to the first occurrence of death from cardiovascular causes, nonfatal myocardial infarction, or nonfatal stroke. The secondary endpoints included cardiovascular composite safety endpoint (time to the first occurrence of death from cardiovascular causes, nonfatal myocardial infarction, nonfatal stroke, or coronary revascularization), venous thromboembolism, or heart failure. These cardiovascular endpoints were adjudicated by an independent clinical events committee whose members were unaware of the group assignments. In addition, based on adverse and serious adverse events data, we analyzed time to the first occurrence of atrial fibrillation and acute kidney injury. COVID-19 status was ascertained by SARS-CoV-2 testing and the diagnosis was extracted from adverse event listings. All efficacy endpoints were prespecified in TRAVERSE substudies, each of which had its own study protocol and statistical analysis plan.

Serum testosterone, dihydrotestosterone, and estradiol levels were measured in an early morning sample after an overnight fast using LC-MS/MS assays that have described previously [17].

### Statistical Methods

Baseline characteristics of study cohort are presented by COVID-19 diagnosis, treatment assignment, and overall. For time-to-event analyses, participants with COVID-19 were followed from the time of the COVID-19 diagnosis and men who were not infected during study were followed from randomization to the end of the study. The Kaplan-Meier curves for MACE, venous thromboembolism, heart failure, atrial fibrillation and acute kidney injury were generated and 1-year Kaplan-Meier estimates were calculated for COVID-19 and non- COVID-19 cohorts. To mitigate against the immortal time bias, the clinical safety events were censored in both the COVID-19 and non-COVID-19 group 1 year after either the COVID-19 diagnosis (for the COVID-19 group) or the randomization (for the NON-COVID-19 group). The difference between the 2 groups was examined using the log-rank test. In addition, Cox Proportional Hazards (Cox PH) models with factor for group (COVID-19 or non- COVID-19) adjusted for prevalent CVD at baseline, treatment assignment, and age were conducted. The hazard ratios and 95% CI were extracted from these models. The comparison of Covid-19 incidence between TRT and placebo groups was performed using Kaplan-Meier estimates and log-rank test.

The comparisons of hormone levels, hemoglobin and hematocrit, and measures of sexual desire, hypogonadal symptoms, energy, and mood between pre- and post-Covid-19 diagnosis, among men who developed COVID-19 after randomization were performed using paired *t* test, adjusting for age and baseline values. Average values from all visits that occurred within 1 year before and after COVID-19 diagnosis (excluding pre-treatment baseline visits) were calculated for each participant. The estimates and corresponding 95% CI and *P* values were provided for each metric by treatment arm. Variables that were assessed at more than one timepoint were compared between COVID-19 and non-COVID-19 groups using mixed effect model regression, adjusting for baseline CVD and age. Sensitivity analyses were performed after excluding the small number of participants who experienced more than one COVID-19 episode.

All hypotheses were tested at the two-sided alpha level of 0.05 and performed using SAS v.9.4 (SAS Institute, Cary, NC). Type 1 error and 95% confidence intervals were not adjusted for multiplicity. As the COVID-19 pandemic was not anticipated, the related analyses were not prespecified nor corrected for multiplicity, and all of the associated *P* values should be considered nominal.

## Results

Among 5204 unique participants who were randomized in the TRAVERSE trial, 379 men—192 in the placebo group and 187 in the testosterone group—had one or more diagnosed episodes of COVID-19 after randomization. Eight men had more than one episode of COVID-19: 6 in the placebo group and 2 in the testosterone group.

### Baseline Characteristics

The characteristics of the study population enrolled in the TRAVERSE trial have been published [16, 17]. Briefly, the average age of the participants was 63 years, 80% self-identified themselves as White, 17% as Black or African Americans, and 16% as Hispanic or Latino. The mean body mass index was 35 kg/m^2^; 54.2% had prior CVD, 75% had diabetes mellitus, and 93% had hypertension.

**Figure 1.**
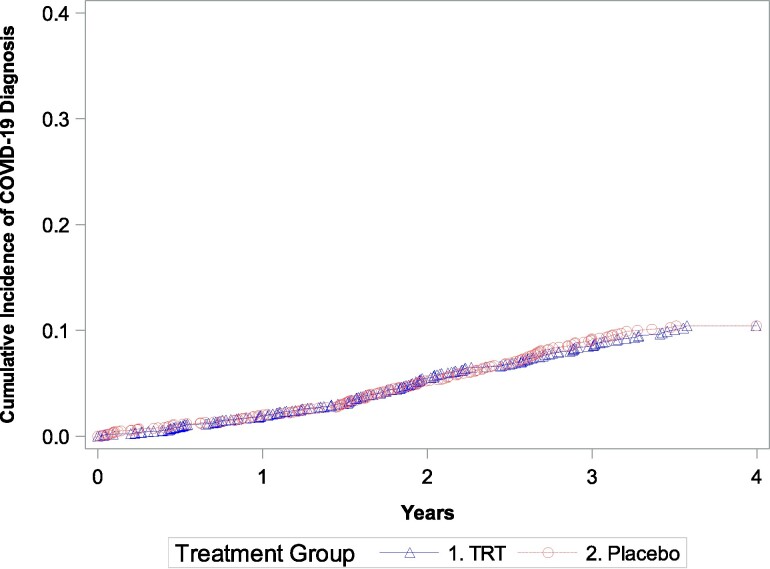
Kaplan-Meier plot showing the cumulative incidence of COVID-19 diagnoses over time since randomization. The 3-year Kaplan-Meier incidence of COVID-19 diagnosis was 8.0% in testosterone group and 8.6% in the placebo arm, *P* = .823.

The baseline characteristics of men who developed COVID-19 were generally similar to those of men who did not (Table 1). The baseline prevalences of CVD (61% vs 55%) and diabetes mellitus (81% vs 75%) were slightly higher in men who developed COVID-19 than in those who did not. Among men who had one or more episodes of COVID-19, the baseline characteristics of men in the TRT and placebo groups were similar (Table 1). The 3-year Kaplan-Meier estimates of the incidence of COVID-19 were similar for TRT (8.0%) and placebo (8.6%) groups (log-rank *P* value = .823) (Fig. 1).

**Table 1.** Baseline characteristics of participants by COVID-19 diagnosis and overall

	Testosterone group (n = 2601)		Placebo group (n = 2603)		All randomized participants (n = 5204)
	Participants without COVID-19 (n = 2414)	Participants with COVID-19 (n = 187)	Participants without COVID-19 (n = 2411)	Participants with COVID-19 (n = 192)	
Age, years	63.4 (7.9) 64.0 (58.0, 69.0)	63.6 (8.5) 64.0 (57.0. 70.0)	63.2 (7.9) 64.0 (58.0, 69.0)	63.7 (7.7) 64.0 (58.0, 70.0)	63.3 (7.9) 64.0 (58.0, 69.0)
Age group					
45 ≤ 65	1263 (52.3%)	97 (51.9%)	1291 (53.6%)	101 (52.6%)	2752 (52.9%)
≥ 65	1151 (47.7%)	90 (48.3%)	1120 (46.4%)	91 (47.4%)	2452 (47.1%)
Current smoker					
Yes	453 (18.8%)	23 (12.3%)	471 (19.5%)	14 (7.3%)	961 (18.5%)
No	1961 (81.2%)	164 (87.7%)	1940 (80.5%)	178 (92.7%)	4243 (81.5%)
Dyslipidemia					
Yes	2168 (89.8%)	176 (94.1%)	2154 (89.3%)	178 (92.7%)	4676 (89.9%)
No	246 (10.2%)	11 (5.9%)	257 (10.7%)	14 (7.3%)	528 (10.2%)
Hypertension					
Yes	2244 (93.0%)	179 (95.7%)	2229 (92.4%)	173 (90.1%)	4825 (92.7%)
No	246 (10.2%)	8 (4.3%)	182 (7.6%)	19 (9.9%)	379 (7.3%)
Prior CV disease					
Yes	1307 (54.1%)	103 (55.1%)	1309 (54.3%)	128 (66.7%)	2847 (54.7%)
No	1107 (45.9%)	84 (44.9%)	1102 (45.7%)	64 (33.3%)	2357 (45.3%)
Prior use of testosterone					
Yes	3 (0.124%)	2 (1.1%)	10 (0.415%)	0 (0%)	15 (0.29%)
No	2411 (99.9%)	185 (98.9%)	2401 (99.6%)	192 (100%)	5189 (99.7%)
Baseline aspirin use					
Yes	1283 (53.1%)	112 (59.9%)	1413 (58.6%)	119 (62.0%)	2774 (53.3%)
No	1131 (46.9%)	75 (40.1%)	998 (41.4%)	73 (38.0%)	2430 (46.7%)
Diabetes mellitus					
Yes	1762 (73.2%)	155 (82.9%)	1811 (75.9%)	152 (79.2%)	3880 (74.7%)
No	646 (26.8%)	32 (17.1%)	532 (22.1%)	40 (20.8%)	1317 (25.3%)
Hematocrit (%)	41.9 (3.8) 42.0 (40.0, 45.0)	41.7 (3.4) 42.0 (40.0, 44.0)	41.9 (3.8) 42.0 (39.0, 45.0)	41.5 (3.8) 42.0 (39.0, 44.0)	41.9 (3.8) 42.0 (40.0, 45.0)
Hemoglobin (g/dL)	14.0 (1.4) 14.1 (13.2, 14.9)	14.0 (1.3) 14.0 (13.0, 14.9)	14.0 (1.3) 14.1 (13.1, 14.9)	13.9 (1.3) 14.0 (13.2, 14.9)	14.0 (1.3) 14.1 (13.2, 14.9)
**Hypogonadism Impact of Symptoms (HIS-Q)**					
HIS-Q total score	46.1 (12.0) 45.7 (38.0, 54.3)	46.1 (11.5) 45.7 (38.0, 54.3)	45.8 (12.1) 45.7 (38.0, 54.3)	46.3 (10.9) 46.7 (39.1, 53.3)	45.9 (12.0) 45.7 (38.0, 54.3)
HIS-Q—Cognition Domain Score	37.0 (17.2) 33.3 (25.0, 50.0)	37.0 (16.4) 33.3 (25.0, 50.0)	36.0 (16.7) 33.3 (25.0, 50.0)	36.7 (17.4) 33.3 (25.0, 50.0)	36.5 (16.9) 33.3 (25.0, 50.0)
HIS-Q—Energy Domain Score	50.6 (23.3) 50.0 (33.3, 66.7)	51.8 (22.7) 58.3 (33.3, 75.0)	50.0 (23.2) 50.0 (33.3, 66.7)	52.0 (21.7) 50.0 (33.3, 75.0)	50.4 (23.2) 50.0 (33.3, 66.7)
HIS-Q—Libido Domain Score	59.1 (18.1) 58.3 (50.0, 75.0)	56.9 (19.8) 58.3 (41.7, 66.7)	59.1 (18.3) 58.3 (50.0, 75.0)	60.1 (17.2) 58.3 (50.0, 75.0)	59.1 (18.2) 58.3 (50.0, 75.0)
HIS-Q—Sexual Function Domain Score	61.3 (20.1) 75.0 (50.0, 75.0)	60.9 (19.6) 75.0 (43.8, 75.0)	60.5 (20.7) 75.0 (43.8, 75.0)	61.4 (19.7) 75.0 (50.0, 75.0)	60.9 (20.3) 75.0 (50.0, 75.0)
HIS-Q—Sleep Domain Score	38.8 (17.9) 41.7 (25.0, 50.0)	41.7 (17.6) 41.7 (33.3, 50.0)	39.3 (18.1) 41.7 (25.0, 50.0)	39.3 (18.5) 37.5 (25.0, 50.0)	39.2 (18.0) 41.7 (25.0, 50.0)

Values are mean (SD), median (IQR) for continuous, and numbers (%) for categorical data. Higher scores on HIS-Q (Hypogonadism Impact of Symptoms Questionnaire) indicate worse performance.

### Baseline and Pre-COVID-19 On-Treatment Hormone Levels in Men Who Had One or More Episodes of COVID-19

Baseline serum total testosterone levels prior to randomization did not differ significantly between men who subsequently developed COVID-19 during the intervention period (mean [SD] 224.4 [46.0] ng/dL) and those who did not (220.0 [46.0] ng/dL, *P* = .297) (Table 2). The incidence of COVID-19 was similar in the TRT and placebo groups (7.2% vs 7.4%, *P* = .808). The baseline testosterone levels prior to treatment initiation were not related to the risk of developing COVID-19.

**Table 2.** Baseline hormone levels in randomized participants with and without COVID-19 and overall

	Testosterone arm (n = 2601)		Placebo arm (n = 2603)		All randomized participants (n = 5204)
	Participants without post-randomization SARS-CoV-2 Infection (n = 2414)	Participants with SARS-CoV-2 infection(s) post-randomization (n = 187)	Participants without post-randomization SARS-CoV-2 infection (n = 2411)	Participants with SARS-CoV-2 infection(s) post-randomization (n = 192)	
**Testosterone**					
Testosterone, ng/dL	220.3 (47.2) 226.9 (218.4, 222.2)	224.0 (43.9) 228.5 (217.7, 230.3)	219.7 (48.1) 225.9 (217.7, 221.6)	224.6 (48.2) 232.3 (217.7, 231.4)	220.4 (47.6) 226.8 (219.1, 221.7)
Testosterone subgroup					
Baseline total testosterone levels < 250 ng/dL	1650 (68.5%)	125 (66.8%)	1645 (68.3%)	126 (65.6%)	3546 (68.2%)
Baseline total testosterone levels ≥ 250 ng/dL	759 (31.5%)	62 (33.2%)	765 (31.7%)	66 (34.4%)	1652 (31.8%)
**DHT**					
DHT, ng/dL	16.2 (8.0) 15.0 (15.9, 16.7)	15.6 (7.6) 13.9 (14.4, 16.8)	16.3 (8.7) 15.1 (15.9, 16.7)	16.9 (7.9) 15.2 (15.7, 18.1)	16.2 (8.3) 15.0 (16.0, 16.4)
**Estradiol**					
Estradiol, pg/mL	20.9 (8.1) 19.9 (20.6, 21.3)	21.8 (8.34) 20.7 (20.5, 23.1)	20.9 (8.4) 19.9 (20.6, 21.3)	20.9 (7.3) 19.6 (19.8, 22.0)	21.0 (8.3) 19.9 (20.7, 21.2)

Values are mean (SD), median (IQR) for continuous, and numbers (%) for categorical data. To convert serum total testosterone concentrations in nanograms per deciliter to nanomoles per liter, multiply testosterone concentration in nanograms per deciliter by 0.0347. To convert estradiol concentrations from picogram per milliliter to picomoles per liter, multiply estradiol concentrations in picogram per milliliter by 3.67. To convert dihydrotestosterone concentrations in nanograms per deciliter to nanomoles per liter, multiply dihydrotestosterone concentrations in nanograms per deciliter by 0.0344.

Abbreviation: DHT, dihydrotestosterone.

**Figure 2.**
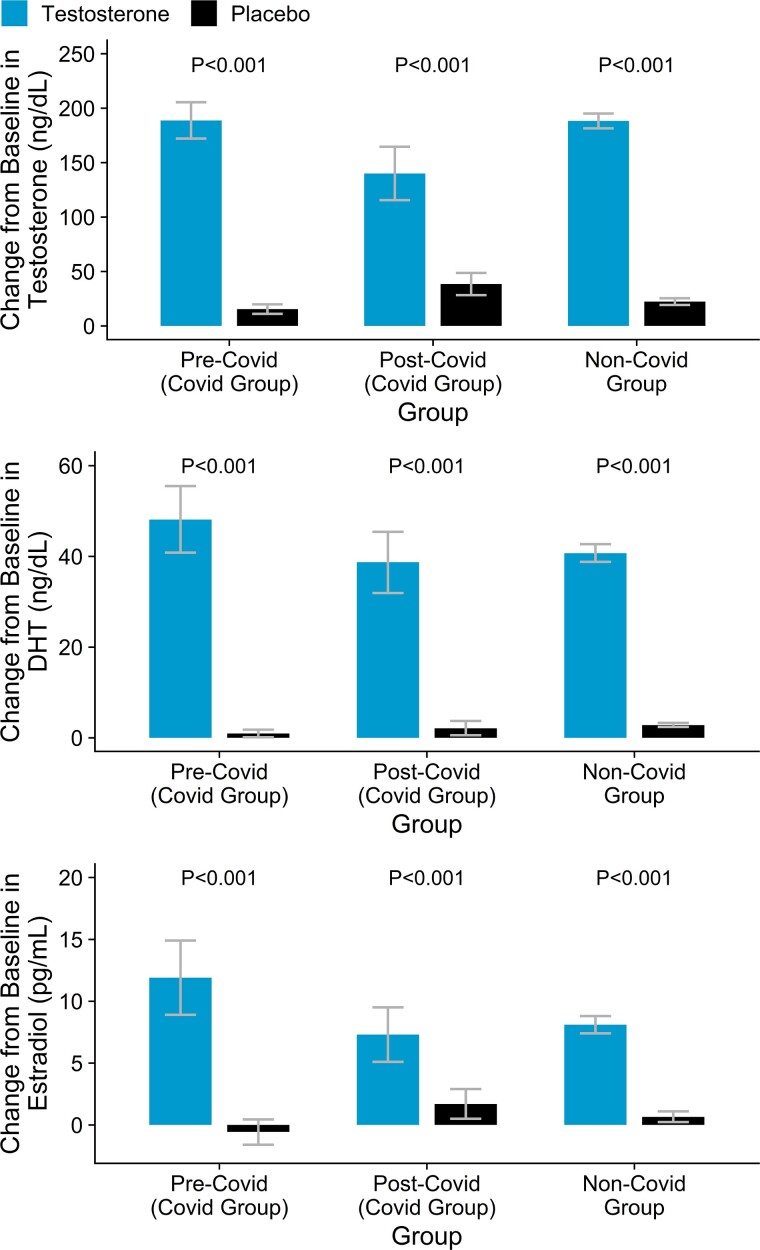
Change from baseline in total testosterone, dihydrotestosterone, and estradiol levels in randomized participants with COVID-19 diagnosis (both before the COVID-19 diagnosis and post-COVID-19 diagnosis) and in participants without the COVID-19 diagnosis by TRT and placebo treatment. Bar graphs for each of the 3 hormones. For COVID-19 Group: change from baseline to on-treatment level in the pre-COVID-19 period in placebo and TRT groups; change from baseline to on-treatment levels during the post COVID-19 period in TRT and placebo group. For NON-COVID-19 Group: change from baseline to mean on-treatment levels during the intervention phase. *P* values for comparison between changes from baseline in treatment and placebo arms were extracted from T test. Abbreviation: DHT, dihydrotestosterone. To convert serum total testosterone concentrations in nanograms per deciliter to nanomoles per liter, multiply testosterone concentration in nanograms per deciliter by 0.0347. To convert estradiol concentrations from picogram per milliliter to picomoles per liter, multiply estradiol concentrations in picogram per milliliter by 3.67. To convert dihydrotestosterone concentrations in nanograms per deciliter to nanomoles per liter, multiply dihydrotestosterone concentrations in nanograms per deciliter by 0.0344.

After randomization, the on-treatment testosterone levels in the testosterone-treated men in the COVID-19 group prior to the COVID-19 diagnosis (411 [376, 445] ng/dL) did not differ significantly from those in the non-COVID-19 group (409 [402, 415] ng/dL, *P* = .898) (Table 3, Fig. 2). Serum testosterone levels in the samples drawn most proximally to COVID-19 diagnoses also did not differ from on-treatment levels in those without COVID-19 diagnoses.

**Table 3.** Changes in hormone levels before and after COVID diagnosis

Hormone	Patients with COVID										
	Testosterone group					Placebo group					*P* value (TRT vs placebo)
	Baseline	Pre-COVID average	Post-COVID average	Difference post − pre	*P* value (pre vs post-COVID-19)	Baseline	Pre-COVID average	Post-COVID average	Difference post − pre	*P* value (pre vs post)	
Testosterone, ng/dL	224.0 (217.7, 230.3) (n = 187)	410.9 (376.3, 445.4) (n = 121)	348.1 (317.3, 378.9) (n = 121)	−62.8 (−101.5, −24.1)	0.002	224.6 (217.7, 231.4) (n = 192)	245.5 (233.2, 257.7) (n = 129)	257.7 (242.4, 273.0) (n = 129)	12.3 (0.888, 23.7)	0.035	<.001
DHT, ng/dL	15.6 (14.4, 16.8) (n = 162)	50.4 (35.4, 65.5) (n = 24)	47.6 (27.5, 67.6) (n = 24)	−2.9 (−21.9, 16.2)	0.758	16.9 (15.7, 18.1) (n = 162)	16.1 (13.2, 19.0) (n = 25)	18.1 (14.3, 21.9) (n = 25)	2.0 (−1.1, 5.1)	0.204	.608
Estradiol, pg/mL	21.8 (20.5, 23.1) (n = 160)	29.4 (23.1, 35.8) (n = 24)	24.2 (19.2, 29.2) (n = 24)	−5.2 (−10.4, −0.090)	0.046	20.9 (19.8, 22.0) (n = 162)	19.1 (16.2, 22.0) (n = 25)	20.6 (17.7, 23.5) (n = 25)	1.5 (−1.3, 4.2)	0.278	.023

	Patients without COVID								
	Testosterone Group				Placebo Group				*P* value (TRT vs Placebo)
	Baseline	Post-baseline average	Difference post-baseline	*P* value	Baseline	Post-baseline average	Difference post-baseline	*P* value	
Testosterone, ng/dL	220.3 (218.4, 222.2) (n = 2393)	408.6 (401.9, 415.3) (n = 2393)	188.3 (181.5, 195.1)	<.001	219.7 (217.7, 221.6) (n = 2389)	242.0 (238.7, 245.4) (n = 2389)	22.4 (19.3, 25.4)	<.001	<.001
DHT, ng/dL	16.2 (15.9, 16.6) (n = 1854)	57.0 (55.0, 59.0) (n = 1854)	40.7 (38.8, 42.7)	<.001	16.3 (15.9, 16.7) (n = 1841)	19.1 (18.6, 19.6) (n = 1841)	2.8 (2.4, 3.3)	<.001	<.001
Estradiol, pg/mL	20.9 (20.6, 21.3) (n = 1837)	29.1 (28.3, 29.8) (n = 1837)	8.1 (7.4, 8.8)	<.001	20.9 (20.5, 21.3) (n = 1827)	21.5 (21.1, 22.0) (n = 1827)	0.646 (0.238, 1.1)	.002	<.001

Pre-COVID and post-COVID means calculated as an average of values recorded up to 365 days before and after COVID-19 diagnosis. To convert serum total testosterone concentrations in nanograms per deciliter to nanomoles per liter, multiply testosterone concentration in nanograms per deciliter by 0.0347. To convert estradiol concentrations from picogram per milliliter to picomoles per liter, multiply estradiol concentrations in picogram per milliliter by 3.67. To convert dihydrotestosterone concentrations in nanograms per deciliter to nanomoles per liter, multiply dihydrotestosterone concentrations in nanograms per deciliter by 0.0344.

Abbreviations: DHT, dihydrotestosterone; TRT, testosterone replacement therapy.

Serum dihydrotestosterone and estradiol levels at baseline and on-treatment prior to the COVID-19 diagnosis were also similar between the men who developed COVID-19 and those who did not (Tables 2 and 3).

### Association of TRT With Disease Severity

The incidences of COVID-19-related hospitalization and deaths as markers of disease severity were similar among those randomized to placebo or the TRT groups: 72 of 187 (38.5%) patients randomized to the TRT group and 63 of 192 (32.8%) patients randomized to the placebo group were hospitalized (Chi-squared test *P* = .222); moreover, 24 of 187 (12.8%) patients randomized to the TRT group and 17 of 192 (8.9%) patients randomized to the placebo group died (*P* = .247). One subject had 2 hospitalizations due to COVID-19 and was counted once.

### Changes in On-Treatment Hormone Levels After COVID-19

Among men in the TRT group who developed COVID-19, serum on-treatment testosterone levels were significantly lower during the year after COVID-19 diagnosis than before COVID-19 diagnosis (change from pre-COVID-19 to post-COVID-19 levels in the TRT group: 63 ng/dL [−102, −24] ng/dL, *P* = .002) (Tables 2 and 3). In contrast, serum testosterone levels in the placebo-treated men increased slightly during the year after COVID-19 (change from pre-COVID-19 to post-COVID-19 levels in the placebo group: difference 12.3 [0.9, 24] ng/dL, *P* = .035). Serum estradiol levels also were significantly lower during the year after COVID-19 diagnosis than before COVID-19 diagnosis in the TRT group (difference −5 [−10, −0.1] pg/mL, *P* = .046) but did not change in the placebo-treated men (Table 3, Fig. 2). On-treatment serum dihydrotestosterone levels did not change significantly in either the TRT or the placebo group after COVID-19 among those who developed COVID-19 (Table 3, Fig. 2).

We considered the possibility that the participants could have stopped the study medication during or for some time after COVID-19 or may have had more limited access to the study medication for some time after testing positive for the virus. Sensitivity analyses that excluded testosterone measurements within 90 days after a COVID-19 diagnosis were performed; these analyses showed similar results (difference between on-treatment testosterone levels prior to COVID-19 diagnosis and testosterone levels after COVID-19 diagnosis: TRT group, −58.3 ng/dL [−103.7, −12.9, *P* = .012]; placebo group, +13.4 ng/dL [95% CI: 0.2, 26.7, *P* = .047]).

### Association Between COVID-19 Infection and Clinical Safety Events

**Figure 3.**
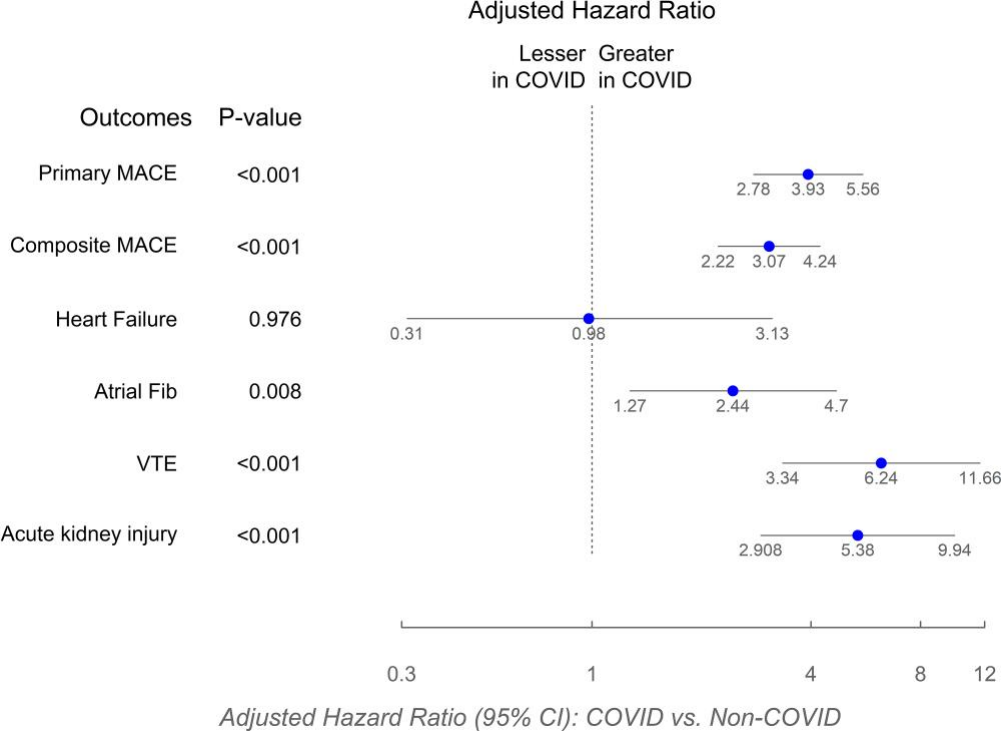
Forest plot of hazard ratios and 95% CI for clinical safety events in COVID-19 and NON-COVID-19 groups overall and by treatment arms (TRT and placebo). Composite MACE is the cardiovascular composite safety event that included revascularization procedures in addition to death due to cardiovascular causes, nonfatal myocardial infarction, and nonfatal stroke. Abbreviations: atrial fib, atrial fibrillation; MACE, major adverse cardiovascular event; VTE, venous thromboembolism.

The COVID-19 diagnosis was associated with a significantly higher incidence of the primary MACE (1-year Kaplan-Meier estimate: 11.1% in men post-COVID-19 vs 2.4% in the non-COVID-19 group, *P* < .001) (Table 4, Fig. 3). Similarly, the incidences of cardiovascular composite safety event that included coronary revascularization procedures (12.8% vs 3.9%, respectively, *P* < .001), and venous thromboembolism events (4.1% vs 0.6%, respectively, *P* < .001) were significantly higher in COVID-19 group compared to the non-COVID-19 group. The incidences of atrial fibrillation and acute kidney injury were also significantly higher in the COVID-19 group compared to the non-COVID-19 group (Table 4, Fig. 3). There was no statistically significant relation between COVID-19 diagnosis and heart failure. The incidences of primary MACE, cardiovascular composite safety event, venous thromboembolism, atrial fibrillation, or acute kidney injury did not differ meaningfully between the testosterone and placebo-treated men who had COVID-19 (Table 4). Sensitivity analysis that excluded participants with more than one COVID-19 infection yielded similar results.

**Table 4.** Clinical safety endpoints among participants with and without COVID-19 (endpoints censored at year 1)

Outcome	Patients with COVID-19	Patients without COVID-19	*P* value (COVID-19 vs non-COVID-19 group)	Patients with COVID-19		Patients without COVID-19	
				Testosterone	Placebo	Testosterone	Placebo
MACE	34/366 11.1%	112/4819 2.4%	<.001	15/187 9.8%	19/192 12.5%	61/2409 2.6%	51/2410 2.2%
Cardiovascular composite safety event	37/353 12.8%	180/4819 3.9%	<.001	16/187 11.0%	21/192 14.7%	98/2409 4.2%	82/2410 3.5%
Heart failure	3/367 1.3%	41/4819 0.9%	.965	1/187 0.9%	2/192 1.6%	22/2409 0.9%	19/2410 0.8%
VTE	13/376 4.1%	28/4819 0.6%	<.001	7/187 4.4%	6/192 3.8%	15/2409 0.6%	13/2410 0.6%
Atrial Fibrillation	6/358 1.9%	58/4816 1.2%	.012	3/178 1.8%	3/180 2.1%	32/2406 1.4%	26/2410 1.1%
AKI	11/358 3.4%	34/4817 0.7%	<.001	8/176 4.8%	3/182 2.1%	18/2407 0.8%	16/2410 0.7%

Values indicate number of cases / total number of participants at risk and 1-year Kaplan-Meier incidence (in %). *P* value for comparison between 1-year Kaplan-Meier rates between all participants with COVID-19 vs all participants without COVID-19 diagnosis. The events in this table were censored at 1 year after randomization (for non-COVID-19 participants and 1 year after COVID-19 diagnosis for COVID-19 cases. Cardiovascular composite safety event included revascularization procedures in addition to death due to cardiovascular causes, nonfatal myocardial infarction, and nonfatal stroke.

Abbreviations: AKI, acute kidney injury; MACE, major adverse cardiovascular event; VTE, venous thromboembolism.

**Figure 4.**
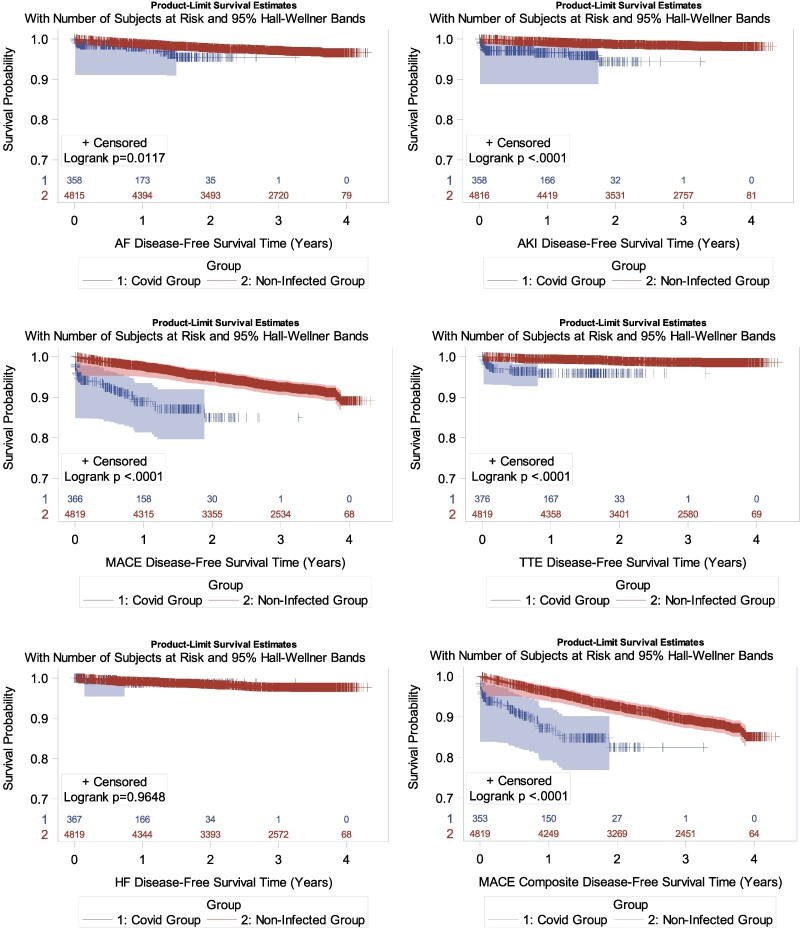
Kaplan-Meier curves for the clinical safety events in the COVID-19 and non-COVID-19 groups during the 1-year after COVID-19 diagnosis in the COVID-19 group and during the 1-year after randomization for the NON-COVID-19 group. The zero time was the date of randomization in the NON-COVID-19 group and the date of COVID-19 diagnosis in the COVID-19 group. The events were censored 1 year after randomization in the NON-COVID-19 group or the COVID-19 diagnosis in the COVID-19 group. The censored events are shown with a + sign. The shaded regions denote the 95% CI.

Sensitivity analyses using Cox proportional hazards models adjusted for preexisting CVD, age, and treatment assignment confirmed significant positive association between COVID-19 diagnosis and primary MACE, cardiovascular composite safety event, venous thromboembolism, atrial fibrillation, and acute kidney injury during the 1 year after randomization for the non-COVID-19 group or during the 1 year after COVID-19 diagnosis (Fig. 4) (hazard ratios, COVID-19 group compared to the non-COVID-19 group: primary MACE 3.93 [2.78, 5.56], *P* < .001; cardiovascular composite safety event that included revascularization procedures 3.07 [2.22, 4.24], *P* < .001; atrial fibrillation 2.44 [1.27, 4.70], *P* < .001; venous thromboembolism 6.24 [3.34, 11.66], *P* < .001; and acute kidney injury 5.38 [5.38 [2.91, 9.94]) (Fig. 4). Heart failure was not significantly associated with COVID-19 diagnosis.

### COVID-19 and Efficacy Outcomes

**Figure 5.**
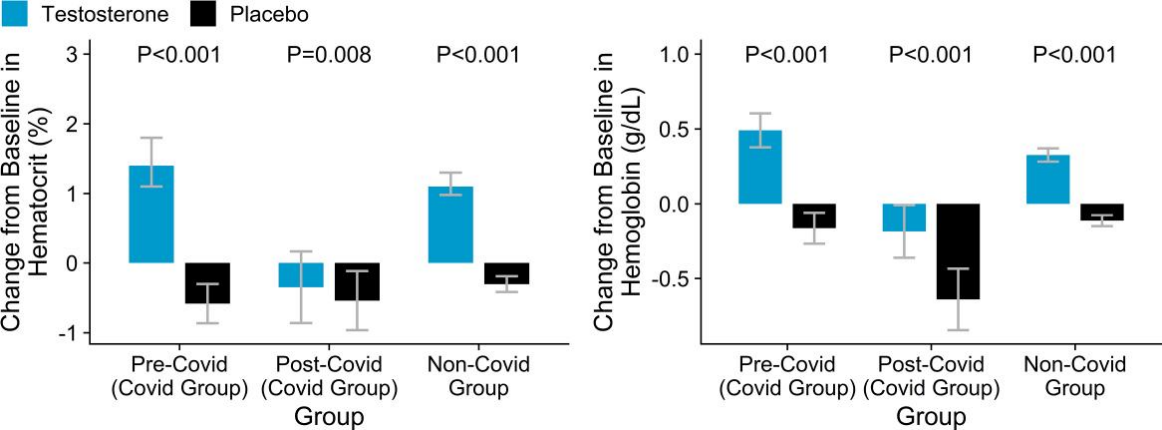
Change from baseline in hemoglobin and hematocrit levels in participants with the COVID-19 diagnosis (both baseline to pre-COVID-19 and baseline to post-COVID-19) and in participants without COVID-19 diagnosis by TRT and placebo treatment. Change from baseline to on-treatment hemoglobin and hematocrit levels in the PRE-COVID-19 period in placebo and TRT groups; change from baseline to on-treatment levels during the post-COVID −19 period in TRT and placebo group. For NON-COVID-19 Group: change from baseline to mean on-treatment levels during the intervention phase. *P* values for comparison between changes from baseline in treatment and placebo arms extracted from T test.

Among participants who had COVID-19, there was a significant decrease in hematocrit (−1.2% [−1.8%, −0.6%]) and hemoglobin levels (−0.49 [−0.73, −0.25] g/dL) in the year after COVID-19 in testosterone-treated men but hematocrit [−0.0% (−0.6%, 0.6%)] and hemoglobin (−0.27 [−0.49, −0.06] g/dL) did not change significantly in the placebo group during the year after COVID-19 compared to pre-COVID-19 period (Fig. 5). In contrast to men who did not have COVID-19 diagnosis and exhibited the expected increase in hemoglobin and hematocrit in the TRT group compared to the placebo group, testosterone-treated men with COVID-19 had a significantly greater decrease in hematocrit relative to placebo-treated men (Fig. 5).

In men without COVID-19, TRT was associated with significantly greater improvements in hypogonadal symptoms, libido, and energy than placebo (Table 5, lower panel). However, in men with COVID-19 diagnosis, the changes from baseline in hypogonadal symptoms, libido, and energy did not differ between the testosterone- and placebo-treated men (Table 5, upper panel). The effects of TRT on hypogonadal symptoms, libido, and energy were significantly smaller in testosterone-treated men with a COVID-19 diagnosis than in testosterone-treated men without COVID-19 (mean between-group difference [95% CI]: hypogonadal symptoms −3.55 [−6.46, −0.63], *P* = .017; libido −2.38 [−3.34, −1.42], *P* < .0001; energy −2.62 [−3.96, −1.28], *P* = .0001).

**Table 5.** Efficacy data before and after COVID-19 diagnosis by treatment arm and baseline and post-randomization averages for randomized participants who did not have COVID-19

Domain	Patients with COVID-19						
	Testosterone group			Placebo group			*P* value (TRT vs placebo
	Pre-COVID average	Post-COVID-19 average	Difference post − pre	Pre-COVID average	Post-COVID-19 average	Difference post − pre	
HIS-Q libido	53.3 (48.6, 58.1)	51.5 (46.5, 56.5)	−1.9 (−5.6, 1.9)	59.1 (53.3, 64.9)	59.6 (53.7, 65.5)	0.524 (−4.6, 5.6)	.450
Energy level	39.7 (33.1, 46.4)	39.8 (33.3, 46.3)	0.1 (−5.9, 6.0)	37.0 (31.0, 42.9)	44.0 (37.3, 50.6)	7.0 (0.5, 13.5)	.117
Mood score	31.1 (27.0, 35.1)	28.5 (24.1, 32.9)	−2.6 (−6.8, 1.6)	29.8 (25.5, 34.0)	33.7 (28.9, 38.5)	3.9 (−0.5, 8.3)	.034
Cognitive score	34.2 (29.8, 38.6)	34.1 (30.1, 38.2)	−0.1 (−4.0, 3.9)	31.6 (27.1, 36.0)	36.4 (31.8, 41.1)	4.9 (1.1, 8.6)	.073
Sexual function	55.0 (48.0, 61.9)	51.8 (45.2, 58.3)	−3.2 (−8.0, 1.6)	60.9 (54.7, 67.2)	61.3 (55.3, 67.4)	0.4 (−5.0, 5.8)	.320
Sleep score	40.0 (35.5, 44.5)	37.1 (32.0, 42.2)	−2.9 (−7.4, 1.7)	41.0 (35.8, 46.2)	42.5 (37.7, 47.4)	1.6 (−2.7, 5.8)	.215
HIS-Q composite	40.4 (37.1, 43.7)	38.5 (35.2, 41.7)	−2.0 (−4.4, 0.478)	41.6 (38.1, 45.2)	44.7 (41.2, 48.3)	3.1 (0.759, 5.4)	.003

	Patients without COVID						
	Testosterone Group			Placebo Group			*P* value (TRT vs Placebo
	Baseline	Post-baseline average	Difference	Baseline	Post-baseline average	Difference	
HIS-Q libido	58.9 (58.1, 59.7)	52.1 (51.4, 52.9)	−6.8 (−7.5, −6.1)	59.2 (58.4, 60.0)	54.8 (54.0, 55.6)	−4.4 (−5.1, −3.8)	<.001
Energy level	50.1 (49.1, 51.1)	39.3 (38.4, 40.1)	−10.9 (−11.8, −9.9)	49.6 (48.6, 50.6)	41.4 (40.4, 42.3)	−8.2 (−9.2, −7.3)	<.001
Mood score	36.3 (35.6, 37.1)	32.4 (31.7, 33.1)	−3.9 (−4.6, −3.3)	36.4 (35.7, 37.2)	33.7 (33.0, 34.4)	−2.8 (−3.4, −2.1)	.012
Cognitive score	36.5 (35.7, 37.2)	32.9 (32.2, 33.5)	−3.6 (−4.3, −3.0)	35.9 (35.2, 36.6)	33.0 (32.4, 33.7)	−2.9 (−3.5, −2.2)	.103
Sexual function	61.0 (60.1, 61.9)	54.7 (53.7, 55.6)	−6.3 (−7.2, −5.4)	60.3 (59.4, 61.3)	56.0 (55.0, 56.9)	−4.4 (−5.3, −3.5)	.002
Sexual desire	60.1 (59.4, 60.8)	53.6 (52.8, 54.3)	−6.5 (−7.2, −5.9)	59.8 (59.1, 60.6)	55.4 (54.7, 56.2)	−4.4 (−5.0, −3.8)	<.001
Sleep score	38.7 (37.9, 39.4)	35.4 (34.7, 36.2)	−3.2 (−3.9, −2.5)	39.2 (38.4, 40.0)	36.8 (36.1, 37.5)	−2.4 (−3.1, −1.7)	.115
HIS-Q composite	45.7 (45.1, 46.2)	40.2 (39.6, 40.7)	−5.5 (−6.0, −5.0)	45.5 (45.0, 46.1)	41.6 (41.1, 42.1)	−3.9 (−4.4, −3.5)	<.001

Pre- and post-Covid-19 means calculated as an average of values recorded up to 365 days before and after COVID-19 diagnosis. Lower scores indicate better function. The negative values for the difference between post and baseline or post-COVID-19 minus pre-COVID-19 scores indicate improvement.

Sensitivity analyses after excluding participants with more than one COVID-19 episode yielded similar results.

## Discussion

Published studies have provided conflicting results on the relationship of hypogonadism with the risk of developing COVID-19. In the TRAVERSE trial, baseline and pre-COVID-19 on-treatment testosterone levels were similar in men who developed COVID-19 and those who did not; furthermore, the incidence of COVID-19 was similar in the testosterone and placebo-treated men. Thus, our analyses of the TRAVERSE trial data indicate that neither baseline testosterone levels nor TRT affects the risk of developing COVID-19. Specifically, hypogonadism was not protective against COVID-19 and TRT did not increase the risk of developing COVID-19.

The occurrence of COVID-19 was associated with substantially increased risk of MACE, venous thromboembolism, atrial fibrillation, and acute kidney injury; the hazard ratios for the risk of venous thromboembolism and acute kidney injury were particularly high. Similar increases in the incidence of MACE, venous thromboembolism, and acute kidney injury in patients with COVID-19 have been reported previously [23]. Although the incidences of MACE, venous thromboembolism, atrial fibrillation, and acute kidney injury did not significantly differ between testosterone-treated and placebo-treated men after the COVID-19 diagnosis, the number of events in subgroups was small and likely did not have sufficient statistical power to evaluate whether there was an interaction between COVID-19 and TRT in increasing the risk of these adverse events. It is possible that the hypercoagulable state associated with COVID-19 could increase the risk of venous thromboembolism in response to TRT.

Serum testosterone and estradiol levels were lower in testosterone-treated men during the year after COVID-19 compared to the on-treatment levels in the same individuals prior to the COVID-19 diagnosis. Low testosterone levels are observed commonly in men with acute infections, including COVID-19 [24, 25], trauma, or acute respiratory failure [26, 27]. However, the reasons for lower testosterone levels in men being treated with testosterone during the year after COVID-19 diagnosis compared to the pre-COVID-19 period while receiving TRT are not clear. Reduced adherence after the COVID-19 diagnosis, increased testosterone clearance, and/or changes in sex hormone binding globulin (SHBG) levels induced by the COVID-19 infection could potentially contribute to lower testosterone levels in the TRT group after the COVID-19 diagnosis. Unfortunately, SHBG levels were not measured. Also, the placebo-treated men after the COVID-19 diagnosis had slightly higher testosterone levels than before the COVID-19 diagnosis, similar to the placebo-treated men without the COVID-19 diagnosis, likely reflecting the regression to the mean phenomenon commonly observed in testosterone trials. Reduced physical or mental ability due to a serious illness such as COVID-19, and access limitation in early days of the COVID-19 pandemic could also have contributed to reduced adherence with the TRT in the COVID-19 group.

Among the placebo-treated participants with COVID-19 diagnosis, hypogonadal symptoms, libido, energy levels, and cognitive scores worsened after the COVID-19 diagnosis. Thus, COVID-19 worsened hypogonadal symptoms, libido, energy level, and cognitive function and appears to have attenuated the effect of TRT on some of the self-reported efficacy domains.

TRT of men with COVID-19 diagnosis was associated with a reduction in hemoglobin and hematocrit levels. This is surprising because testosterone treatment has been shown to improve anemia of inflammation [28] and because the placebo-treated men with COVID-19 had only a minimal change in their hemoglobin and hematocrit levels during the year after COVID-19 diagnosis. The mechanisms by which COVID-19 attenuates erythropoietic response to TRT are not known. Severe systemic inflammatory response associated with infections such as COVID-19 may limit iron availability for erythropoiesis [29]. We have shown that iron availability plays an important role in mediating testosterone's effect on erythropoiesis [30] and that in an iron-restricted mice, testosterone treatment worsens anemia because of ineffective erythropoiesis possibly due to erythropoietin resistance [30]. Thus, it is possible that restricted iron availability due to the severe inflammatory and cytokine response associated with COVID-19 could lead to a reduced erythropoietic response to TRT.

TRAVERSE is the first randomized trial to evaluate the risk of COVID-19 infection in men with hypogonadism and the effect of COVID-19 on the response to TRT. Unlike previous reports that have utilized retrospective analyses of electronic medical records or insurance data, the TRAVERSE study was a prospective randomized, placebo-controlled trial. Testosterone levels in TRAVERSE were measured using an LC-MS/MS assay, in a laboratory certified by the Center for Disease Control and Prevention's Hormone Standardization Program for Testosterone. The safety, as well as efficacy, endpoints were assessed using prespecified structured protocols.

One limitation of the study is that the allocation to COVID-19 and non-COVID-19 groups was not randomized. We attempted to mitigate the immortal time bias associated with the different times of COVID-19 diagnoses by calculating the safety event rates during the 1 year after randomization for the non-COVID-19 group and during the 1-year after COVID-19 diagnosis for the COVID-19 group, thus equalizing the time duration during which events took place. However, those time periods are not concurrent. The analyses reported here were not prespecified because the COVID-19 pandemic was not anticipated; therefore, these findings need further confirmation. The CI and type 1 errors were not adjusted for multiplicity. The numbers of some of the safety events (eg, venous thromboembolism and acute kidney injury) were small and likely did not have sufficient statistical power for meaningful comparison of the incidence in the testosterone- and placebo-treated men in the COVID-19 group. Similarly, the statistical power for the analyses of the testosterone's treatment effect on some of the efficacy outcomes may have been limited. Because study participants were not systematically tested for COVID-19, asymptomatic or mildly symptomatic participants with SARS-CoV-2 infection were likely not detected. Serum samples were not available for assays of luteinizing hormone, follicle stimulating hormone, SHBG, and other hormones to investigate the mechanisms by which COVID-19 affects the hypothalamic-pituitary-testicular axis. The impact of COVID-19 infection on the recovery of reproductive function after cessation of prolonged exogenous testosterone treatment was not evaluated. By virtue of the trial's eligibility criteria, the study enrolled men with hypogonadism and preexisting or increased risk for CVD; therefore, wider extrapolation to the general population should be constrained.

## Conclusions

In men with hypogonadism with or at increased risk of CVD, baseline and pre-COVID-19 on-treatment testosterone, dihydrotestosterone, and estradiol levels were similar in those who developed COVID-19 and those who did not. The incidences of COVID-19 diagnoses, and COVID-19-related hospitalizations and deaths, were similar in the TRT and placebo groups. The occurrence of COVID-19 was associated with attenuation of the treatment response to TRT and a reduction of hemoglobin and hematocrit with testosterone treatment.

## Data Availability

Original data generated and analyzed during this study are included in this published article. Some datasets generated during and/or analyzed during the current study are not publicly available but are available from the corresponding author on reasonable request.

## Clinical Trial Information

ClinicalTrials.gov Identifier: NCT03518034.

## Abbreviations

ACE2: angiotensin converting enzyme 2
ADT: androgen deprivation therapy
CVD: cardiovascular disease
DHT: dihydrotestosterone
LC-MS/MS: liquid chromatography tandem–mass spectrometry
MACE: major adverse cardiovascular events
SARS-CoV-2: severe acute respiratory syndrome coronavirus 2
SHBG: sex hormone binding globulin
TRT: testosterone replacement therapy
